# Socio-Demographic and Anthropometric Findings of Women Caregivers in Qwa-Qwa, Free State Province, South Africa

**DOI:** 10.3390/nu18121898

**Published:** 2026-06-11

**Authors:** Queen E. M. Mangwane, Abdulkadir Egal, Delia Oosthuizen

**Affiliations:** 1 Department of Hospitality Management, Faculty of Management Sciences, Tshwane University of Technology, Pretoria 0001, South Africa; 2 Department of Tourism & Integrated Communication, Faculty of Human Sciences, Vaal University of Technology, Vanderbijlpark 1900, South Africa; egal@ieaso.so (A.E.); deliao@vut.ac.za (D.O.)

**Keywords:** women caregivers, anthropometric status, socioeconomic vulnerability, malnutrition

## Abstract

**Background:** Women remain the primary caregivers globally, especially in rural, low-resource settings plagued by poverty, unemployment, low education and poor infrastructure. These factors limit caregiving capacity, heighten vulnerability and increase the risk of food insecurity in female-headed households. **Objective:** To establish a baseline profile of caregivers of primary school children. **Methods:** Phase 1 (baseline) of the study was conducted using a quantitative, exploratory cross-sectional survey design among 75 female caregivers of children aged 7–13 years in Qwa-Qwa, Free State Province. Participants were recruited using convenience sampling. Data were collected with a structured, pre-validated questionnaire on socio-demographics, alongside anthropometric measurements. Data were analysed using descriptive statistics. **Results:** Most participants were unemployed (73.3%) and had low educational attainment, with 86.7% having completed primary school or less. A substantial proportion of households (80.0%) reported a monthly income below R1000. Food insecurity was common, with 69.3% of caregivers reporting experiences of food shortages. Household infrastructure was limited, particularly in refuse removal services (96.0% without access). Despite these socio-economic constraints, a high prevalence of overweight and obesity (72.5%) was observed amongst the participants. **Conclusions:** Caregivers experience severe, overlapping socio-economic and environmental vulnerabilities alongside a high prevalence of overweight and obesity. The study highlights the need for multi-sectoral interventions focused on poverty reduction, rural infrastructure development, improved service delivery, women’s empowerment and strengthened livelihood opportunities to improve household nutrition and resilience.

## 1. Introduction

Caregivers are individuals who perform a specific role for others, whether for a child or adult [1], sometimes an external individual, family members or parents [2]. The caregiving role involves assistance with hygiene and emotional support [3,4], nurturing care, verbal and physical interaction [5], and in many instances, provision of food, including purchasing, preparing, serving and feeding [3,6,7,8]. Many parents rely on external caregivers to assist with their children, especially after school and when they have to work or seek financial stability [9,10]. When children spend extended periods of time with caregivers, the caregivers’ behaviour may play an important role in shaping the quality of life and influencing long-term behaviours [6,11,12,13]. The level of influence is, however, affected by variables such as the practices, knowledge and beliefs, physical health and available resources, which also affect the ability to perform the role successfully. In addition, cultural differences, workload and availability of time, family and social support are linked to the overall nutritional status and well-being of those being assisted [5,14].

Women continue to undertake the majority of unpaid and family caregiving responsibilities. According to recent estimates, women make up about 61% of caregivers, and they continue to provide significantly more unpaid care and household labour than men do [15,16,17]. These results demonstrate how caregiving is still gendered in a variety of contexts. Despite ongoing socioeconomic challenges and a lack of formal support networks, women often take on primary caregiving obligations in rural and low- and middle-income contexts [7,18].

Globally, women’s social and economic position is increasingly recognised as a critical determinant of household nutrition and food security outcomes [19,20]. Women with higher levels of educational attainment and improved access to income-generating opportunities are better positioned to secure diverse and nutritious diets and to reduce the risk of malnutrition within their households. In contrast, women with low literacy levels and limited economic opportunities often struggle to implement effective caregiving practices, even when they possess some degree of nutrition knowledge, underscoring that knowledge alone is insufficient to improve nutrition outcomes in contexts where material resources are scarce [21].

Despite the age of the dataset (2013), the findings remain relevant because the structural determinants identified continue to characterise many rural South African communities. Recent evidence continues to show that socio-economic inequalities remain one of the strongest predictors of household dietary diversity and child nutrition outcomes in low- and middle-income settings [22]. This challenge is particularly pronounced in rural communities, where caregiving roles are predominantly assumed by women who experience multiple, interrelated vulnerabilities, including poverty, unemployment, food insecurity and limited educational attainment, all of which constrain their capacity to provide adequate care for children and other dependents [1,18,23]. These constraints are further intensified by structural barriers such as poor infrastructure, inadequate sanitation services and limited access to safe water, which exacerbate health and nutrition challenges in resource-poor settings [24]. Despite global development efforts, rural poverty and service inequities remain unchanged in many sub-Saharan African contexts, continuing to shape caregiving capacity and household nutrition outcomes [25]. Consequently, rural women often shoulder a disproportionate burden of caregiving responsibilities in environments characterised by inadequate household resources and minimal external support, where limited livelihood opportunities and restricted access to resources create precarious living conditions and place additional strain on their ability to balance food provision, childcare and household wellbeing [26,27,28]. More recent national survey data confirm that female-headed households in South Africa continue to experience significantly higher rates of food insecurity and poverty compared to male-headed households [28,29].

This paper reports on the baseline (phase 1) of a larger Nutrition Education Programme (NEP) intervention and was designed to establish an overall baseline profile of caregivers before implementation of the intervention, as well as to serve as a reference point for comparison with post-intervention outcomes. Establishing a baseline profile of the caregivers is an important starting point when planning interventions, especially nutrition education interventions. The socio-demographic, economic and anthropometric characteristics can influence not only nutrition knowledge, but also purchasing behaviour and the ability to implement sustainable strategies, especially when guidance is provided. Additionally, the mode of intervention is also impacted by educational and cultural background. Studies have highlighted how variables such as educational level, household income, living conditions and nutritional status may impact dietary practices and health outcomes of other members or dependents [30,31]. Baseline profiling, therefore, provides a basis for interpreting whether the success of an intervention can be measured. Furthermore, the anthropometric indices can give insight into the nutritional vulnerability and health status of the participants and help understand the possible factors influencing the ability to care for others [32].

Despite growing research on food insecurity and malnutrition in South Africa, limited evidence exists on the socio-demographic, environmental and nutritional realities of women caregivers in under-resourced rural communities. This study contributes baseline evidence from Qwa-Qwa, Free State Province, highlighting the intersecting burdens of poverty, food insecurity and overweight/obesity among caregivers. The findings provide important contextual insight for nutrition intervention planning, rural health policy and future longitudinal caregiver research.

## 2. Materials and Methods

The broader study examined the impact of nutrition education on caregivers’ nutrition knowledge, dietary diversity, food intake patterns and food choice behaviours, reported elsewhere [33]. The overall project was guided by the Nutrition Education Programme (NEP) framework developed by the Food and Agriculture Organization [13], which was adapted and implemented in four sequential phases. The first phase (baseline assessment) focused on establishing the socio-economic and anthropometric characteristics of caregivers, providing a comprehensive overview of existing conditions against which the effects of the NEP intervention could be assessed. This phase also aimed to identify vulnerabilities among caregivers, particularly women in rural households. Their socio-economic conditions and nutritional status may influence household nutrition practices and participation in intervention activities. The research was conducted in a rural community in Qwa-Qwa, Free State province, South Africa. The area is characterised by mountainous terrain, geographic isolation, high population density, widespread poverty and limited economic opportunities [34]. Many households rely on subsistence agriculture and social support due to high unemployment and poor infrastructure. Female-headed households are common, and many women engage in informal income-generating activities to support their families [34]. The selection of Qwa-Qwa was based on the Integrated Nutrition Project implemented by the Centre for Sustainable Livelihoods at the Vaal University of Technology, which targeted rural communities vulnerable to food insecurity and nutrition-related challenges among school-aged children and their caregivers. Women caregivers of children attending the selected primary school were included because the children had participated in the previous study. The follow-up aimed to reinforce the earlier intervention and promote positive nutrition behaviours among children. A quantitative, exploratory cross-sectional survey design was used to assess the socio-demographic characteristics and nutritional status of women caregivers of primary school children aged 7–13 years residing in the village. The village was purposively selected based on its rural context, high levels of poverty and food insecurity and prior participation in a nutrition education initiative. Women caregivers were targeted due to their role in household food provision and nutrition-related decision-making. The exclusion criteria included male caregivers and caregivers who were not responsible for primary school children aged 7–13 years attending the selected school. A sample size calculation formula was used to determine the number of subjects needed for statistical significance. Based on a 95% confidence level, the calculation indicated that 70 subjects were needed. For phase 1, 109 caregivers initially signed informed consent, whereby 96 were recruited for the baseline assessment. Unfortunately, 21 of those did not return for the Phase 1 data collection. This left a final baseline sample of 75, which still met the required sampling power (*n* = 70). Data collection tools included a pre-validated socio-demographic questionnaire [35] administered in the participants’ preferred language (Sesotho), as well as anthropometric measurements of body weight and height, to determine nutritional status using body mass index (BMI) classifications. The socio-demographic questionnaire captured information on participants’ environmental and living conditions. The socio-demographic questionnaire had been previously piloted and applied in studies conducted among the same population and was subsequently pretested during fieldworker training for the current study. Participation was voluntary, and all participants provided written informed consent after the study objectives and procedures were explained. Ethical approval for the broader project was granted by the University of the Witwatersrand Medical Ethics Committee for Research on Human Beings (M080931), and the study was conducted in accordance with the ethical guidelines of the South African Medical Research Council (MRC). Additional permission to conduct the study was obtained from relevant community leadership structures. Data collection for the baseline assessment was conducted during October 2013. Descriptive statistics were used to summarise socio-demographic characteristics and anthropometric indicators, including frequencies and percentages where appropriate. No stratified analyses were conducted for BMI against other variables. The only stratified analyses were based on sociodemographic variables and dietary quality, reported elsewhere.

## 3. Results

### 3.1. Socio-Demographic Profile

Table 1 summarises the socio-demographic characteristics of the respondents. Most participants were unemployed (73.3%, *n* = 55) and almost all reported Sesotho as their primary home language (93.3%, *n* = 70). Nearly half of the participants were married (48.0%, *n* = 36), while just over one-third were single (36.0%, *n* = 27). A smaller proportion were widowed (13.3%, *n* = 10) and only a few were divorced (2.7%, *n* = 2). Educational attainment was low, with the majority having completed only primary school (70.7%, *n* = 53) or reporting no formal education (16.0%, *n* = 12). Almost half of the respondents (45.3%, *n* = 34) reported a monthly income below R500, while 34.7% (*n* = 26) reported a monthly income of between R501 and R1000. A small proportion (6.7%, *n* = 5) reported income above R2000 per month.

### 3.2. Household Environment and Infrastructure

Most households accessed water from a tap located in the yard (80.0%, *n* = 60), while a few had a tap located inside the house (18.7%, *n* = 14), as shown in Table 2. The household infrastructure of the participants indicates that most participants resided in brick houses (78.7%, *n* = 59), while a smaller proportion resided in zinc structures or informal shacks (21.3%, *n* = 16). Regarding sanitation facilities, 89.3% (*n* = 67) of households used pit latrines, while 10.7% (*n* = 8) had access to flush toilets. Access to refuse removal services was limited, as almost all participants reported no formal refuse removal services (96.0%, *n* = 72). Half of the participants (54.7%, *n* = 41) had electricity as a cooking fuel, while 34.7% (*n* = 26) relied on paraffin.

Table 3 presents household food procurement and decision-making practices among the caregivers. Most participants reported purchasing food from spaza shops (85.3%), with few obtaining food from supermarkets (13.5%) or street vendors (1.2%). The majority of households purchased food once a month (84.0%), while 16.0% (*n* = 12) purchased food weekly or daily. Mothers were primarily responsible for food-related decisions, including determining the type of food purchased (68.6%) and the amount spent on food (60.0%). Mothers were also the main caregivers responsible for food preparation (74.3%) and feeding children (77.1%). Overall, food procurement, decision-making, and caregiving responsibilities were predominantly undertaken by mothers within the household.

Table 4 presents indicators of household food availability. Most caregivers reported consuming two meals per day (71.4%; *n* = 53). In addition, a substantial proportion of caregivers (69.3%; *n* = 52) reported experiencing food shortages at varying frequencies (always, often, sometimes, or seldom).

### 3.3. Anthropometric Measurements (Nutritional Status)

Weight was measured using a calibrated digital scale to the nearest 0.1 kg, and height was measured using a stadiometer to the nearest 0.1 cm. Participants were measured barefoot and wearing light clothing. BMI was calculated using the World Health Organization (WHO) equation (BMI = weight in kilograms divided by height in metres squared, kg/m^2^). Participants were classified according to WHO criteria as underweight (<18.5 kg/m^2^), normal weight (18.5–24.99 kg/m^2^), overweight (25.0–29.99 kg/m^2^), or obese (≥30.0 kg/m^2^). Among the 75 participants (see Table 5), 23.5% (*n* = 18) were classified as having normal weight. Notably, a higher proportion of participants were classified as overweight (33.0%) while 39.5% were classified as obese across obesity classes I, II, and III, indicating a high prevalence of excess body weight within the study population.

## 4. Discussion

### 4.1. Socio-Economic Vulnerability and Food Insecurity Among Caregivers

The socio-economic profile of caregivers in rural communities shows a consistent pattern across multiple studies [36,37,38,39,40]. In a study of 184 caregiver–child pairs in the Eastern Cape, a high prevalence of household food insecurity was reported among caregivers, even in households with access to basic services such as tap water and flush toilets [38]. Similarly, findings from rural Limpopo highlighted that knowledge deficiencies and limited caregiver capacity impacted their ability to provide adequate nutrition for young children [39]. Similar disparities were evident in KwaZulu-Natal [41], where nutrition knowledge gaps were evident among caregivers. In rural Mpumalanga, caregivers experienced significantly higher levels of food insecurity compared to non-caregivers, reinforcing the vulnerability associated with caregiving roles in resource-limited rural environments [40]. Across the African continent, limited resources and restricted market access continue to place both physical and emotional strain on caregivers. A study from Southeast Nigeria showed that even where infrastructure (e.g., electricity and safe water) is available, poor market access constrained caregivers’ ability to provide diverse diets [42]. Similar findings were reported in Tanzania and South Africa, where poverty and low income limited dietary variety and access to nutritious foods [43,44]. These realities were also evident within this study community, where most participants relied on spaza shops, which often offer limited fresh produce options.

### 4.2. Education, Income and Caregiver Capacity

Within the rural Qwa-Qwa study population, women caregivers displayed a highly disadvantaged socio-economic profile, characterised by low educational attainment, high unemployment and limited household income. Many caregivers were living on less than R500 per month (45.3%), which remains below the extreme poverty line of R777 per person per month reported in 2023, more than a decade later [45]. With many rural households being female-headed, a significant risk of difficulty is placed on both caregiving and financial responsibilities. Similar demographic patterns were reported in another South African province, where most participants were women with comparable marital profiles [41]. Low socio-economic status may influence caregiver capacity and their ability to provide optimal care. Household socio-economic conditions significantly influence child nutrition outcomes [46]. Education and economic stability, together with access to resources, play a key role in determining the quality of caregiving and influencing child health outcomes. Low educational attainment in this study was particularly concerning, with 16.0% of caregivers having no formal education and 38.8% having only primary school education. Low education levels have been consistently associated with inadequate nutrition knowledge, suboptimal dietary practices and reduced ability to make informed health decisions [47,48,49,50,51]. Supporting this, research in Malawi showed an association between caregiver nutritional status and the home environment, stressing the importance of caregiver wellbeing in child nutrition interventions [52].

### 4.3. Poverty, Food Purchasing Patterns and the Nutrition Transition

Socio-economic constraints strongly influence food access, dietary choices and overall household nutrition [29,32]. Financial limitations often lead households to rely on inexpensive staple foods such as maize meal and fats purchased from spaza shops. While affordable, these foods are often described as nutrient-poor and may be associated with lower dietary diversity and an increased risk of overnutrition alongside resource-constrained settings [32]. Results from resource-limited settings also show that caregivers frequently purchase food on credit or borrow food due to insufficient funds, reflecting persistent economic vulnerability. These patterns align with the global nutrition transition observed in low-income populations, where poverty and limited food access drive consumption of energy-dense, nutrient-poor foods [53]. Individuals living in socio-economic disadvantage are particularly vulnerable to obesity. In this study, low education, unemployment and limited income coincided with elevated BMI levels, consistent with findings from other studies [32,38,51]. The coexistence of high levels of caregiver overweight/obesity and socio-economic vulnerability observed in this study may reflect the complex nutritional challenges reported in similar low-resource settings. Overweight and obesity prevalence reached 72.5%, consistent with national findings [32,33]. To better understand obesity, which is not necessarily associated with total food intake, a study demonstrated that the continuous intake of cheap, energy-dense food can alter biological changes resulting in adverse health effects [54]. The high prevalence of obesity observed among caregivers in the Qwa-Qwa community may reflect the broader socio-economic and environmental challenges described in the literature. However, dietary quality and health outcomes were not directly assessed in the present analysis, and therefore, no conclusions can be drawn regarding the underlying causes of obesity or its long-term health implications.

### 4.4. Gender Roles, Caregiving Burden and Household Wellbeing

Women carried the primary responsibility for household food decisions (88.0%) and meal preparation (89.3%). However, most had education levels below secondary schooling, reflecting the gendered burden of caregiving. High responsibility combined with limited education may influence caregivers’ access to nutrition information and the ability to implement recommended dietary practices [23,26]. Additional pressures arise from poor household living conditions. Studies in low-income Cape Town communities demonstrated that poor household hygiene can increase caregiver stress and health risks, adding to existing socio-economic burdens [55].

### 4.5. Environmental Conditions and Health Risk

Household environmental conditions further compound health risks. Most participants relied on pit latrines and lacked formal refuse removal services. Similar patterns were observed in other South African rural communities, where many households lacked in-home water access and relied on pit latrines. In KwaZulu-Natal, just over half of households had access to safe drinking water and clean cooking fuel, both strongly linked to caregiver wellbeing [41]. Poor sanitation and waste management increase susceptibility to infectious diseases and may worsen nutritional vulnerability [56,57,58]. When combined with poverty, these environmental challenges reduce caregivers’ ability to provide optimal nurturing care [1].

### 4.6. Caregiver Anthropometry and Nutritional Vulnerability

Studies completed in Mpumalanga and the Free State show a high prevalence of caregiver overweight and obesity, often ranging between 45% and 55% and exceeding 66%, respectively, across the provinces [51,59]. Similarly, a high prevalence of overweight or obese caregivers was reported in the Eastern Cape, showing the link between caregiver nutritional status and child growth outcomes [38]. Emerging research shows that caregiver anthropometry is strongly associated with child nutrition outcomes [1,5,60]. Mothers are often the primary caregivers who remain responsible for child feeding, nutrition provision and overall care practices [2]. In rural Limpopo, research reported the coexistence of maternal overweight and child undernutrition within the same households, illustrating the complexity of nutritional challenges experienced by vulnerable communities [2]. Recent reports [54] show that a high BMI in low-resource settings may be associated with adverse health outcomes, although health status was not assessed in the present study. This implies that both under- and overnutrition can arise from within the same environment. Rural populations remain vulnerable to nutrition risks related to poverty, food insecurity and limited dietary diversity [59]. Overall, caregiver anthropometry should be interpreted within a broader socio-ecological context where poverty, food environments, infrastructure limitations and gendered caregiving roles may influence nutritional status. These patterns are consistent with other rural South African settings, where female caregivers, particularly mothers, frequently exhibit a high prevalence of overweight and obesity despite living in resource-constrained and food-insecure households, while child undernutrition may also persist.

## 5. Conclusions

In conclusion, the baseline results of the study show that women caregivers in rural Qwa-Qwa have significant nutritional, environmental and socio-demographic risks. These are typified by severe income constraints, low educational attainment and high unemployment rates, frequently in households led by women. Anthropometric results indicate a high prevalence of overweight and obesity alongside reports of family food shortages and limited municipal service delivery. The findings demonstrate that household food access and nutrition outcomes may be influenced by broader contextual factors, including access to local markets, poor infrastructure and varying food prices. The findings identify several socio-economic and environmental factors that warrant further investigation and consideration in future interventions.

Given the descriptive and cross-sectional nature of the study, the findings should be interpreted as exploratory and hypothesis-generating rather than indicative of causal relationships. Nevertheless, the results provide useful baseline information and identify several socio-economic and environmental factors that may warrant further investigation in future studies examining caregiver nutrition and household food security in rural South African communities.

## 6. Limitations

The first limitation concerns the relatively small sample size (*n* = 75), which may have reduced the study’s statistical power and limited the ability to capture variability within the target population. Secondly, the study employed a cross-sectional, exploratory baseline design, which restricts interpretation to associations at a single point in time. Consequently, the findings are limited to descriptive observations and cannot establish causal relationships or assess changes over time. The study was also conducted within a single rural, resource-constrained community in Qwa-Qwa, a context-specific setting, characterised by particular socio-economic and environmental conditions, which limits the external validity of the findings and their applicability to other settings, including urban or more socio-economically diverse populations. A further limitation is that the sample comprised only women caregivers. While this focus is supported by evidence highlighting women’s central role in household food and caregiving practices33, the exclusion of male caregivers limits the scope of the findings. The results cannot be generalised to men or to mixed-gender household decision-making contexts and potential gender differences were not explored. Overall, these limitations should be considered when interpreting the findings, which should be viewed as exploratory and hypothesis-generating.

## 7. Recommendations

Based on the baseline findings, the following recommendations are proposed for consideration by future researchers and policymakers:

- The baseline results highlight the need for policies that create employment opportunities, particularly for women’s empowerment.
- Increase investment in socio-economic infrastructure within rural areas to enhance living conditions and support community development.
- Sustainable food security and income-generation programmes should be developed to improve household food security and economic resilience.
- Integration of nutrition education within social protection programmes should be explored to address malnutrition and promote healthy dietary practices, especially in areas without existing interventions.
- Improvements to the rural food environment are necessary to increase access to affordable, nutritious foods.
- Implementation of women’s empowerment initiatives is essential, particularly those that enhance decision-making, education and economic participation.
- Primary healthcare services should include structured nutrition counselling to support caregivers and promote optimal nutrition practices at the household level.
- The dataset generated from this study presents opportunities for future research focusing on food insecurity, poverty, overweight and obesity among caregivers, particularly within rural South African contexts.

## Figures and Tables

**Table 1:** Socio-Economic Indicators (*n* = 75).

Variable	Category	Frequency (*n*)	Percentage (%)
Marital status	Single	27	36.0
	Married	36	48.0
	Widowed	10	13.3
	Divorced	2	2.7
Education level	No education	12	16.0
	Primary school	53	70.7
	Secondary school	3	4.0
	College	7	9.3
Monthly income	<R500	34	45.3
	R501–R1000	26	34.7
	R1001–R2000	10	13.3
	>R2000	5	6.7
Home language	Sotho	70	93.3
	Xhosa	1	1.3
	Zulu	2	2.7
	Other	2	2.7

**Table 2:** Household infrastructure.

Variable	Category	Frequency (*n*)	Percentage (%)
Type of house	Brick house	59	78.7
	Informal dwelling (Shack)	16	21.3
Sanitation	Pit latrine	67	89.3
	Flush toilet	8	10.7
Refuse removal	None (Own dump)	72	96.0
	Municipal removal	3	4.0
Fuel for cooking	Electricity	41	54.7
	Paraffin	26	34.7
	Gas/Wood	8	10.7
Source of water	Tap inside the house	14	18.7
	Tap outside the house (in yard)	60	80.0
	Fetch water from elsewhere	1	1.3

**Table 3:** Food purchasing behaviour of caregivers.

Variable	Category	Frequency (*n*)	Percentage (%)
Food source	Spaza shop	64	85.3
	Supermarket	10	13.5
	Street Vendor	1	1.2
Frequency of purchasing	Once a month	63	84.0
	Weekly/daily	12	16.0
Decision on the type of food purchased	Mother	51	68.6
	Grandmother	13	17.1
	Child	4	5.7
	Other	6	8.6
Decision on how much is spent on food	Mother	45	60.0
	Grandmother	13	17.1
	Grandfather	2	2.9
	Father	13	17.1
Responsible for food preparation	Mother	56	74.3
	Grandmother	11	14.3
	Child	2	2.9
	Grandfather	11	14.3
	Other	2	2.9
Responsible for feeding the child(ren)	Mother	58	77.1
	Grandmother	9	11.4
	Child	4	5.7
	Grandfather	2	2.9
	Father	2	2.9

**Table 4:** Caregivers’ Household Food Situation.

Variable	Category	Frequency (*n*)	Percentage (%)
Food shortage	Always	11	14.6
	Often	8	10.7
	Sometimes	32	42.7
	Seldom	1	1.3
	Never	23	30.7
Meals per day	1 meal	20	25.9
	2 meals	53	71.4
	3 meals	2	2.7

**Table 5:** Anthropometric Profile of Caregivers.

BMI Category	Range (kg/m^2^)	Frequency (*n*)	Percentage (%)
Underweight	<18.5	3	4.0
Normal weight	18.5–24.9	18	23.5
Overweight	25.0–29.9	24	33.0
Obese 1	30.0–34.99	20	26.5
Obese 2	35.0–39.99	5	6.5
Obese 3	>40.0	5	6.5

## Data Availability

The data supporting the findings of this study are available upon reasonable request from the corresponding author, mangwaneqem@tut.ac.za. Access to the data will be provided in accordance with ethical guidelines and institutional policies.
